# Supplementary material for: Optimal minimal residual disease threshold in pediatric acute myeloid leukemia: A retrospective cohort study based on the TARGET database
Source: PLoS Med. 2026 May 8;23(5):e1005088. doi: 10.1371/journal.pmed.1005088 (PMC13155632; doi:10.1371/journal.pmed.1005088)

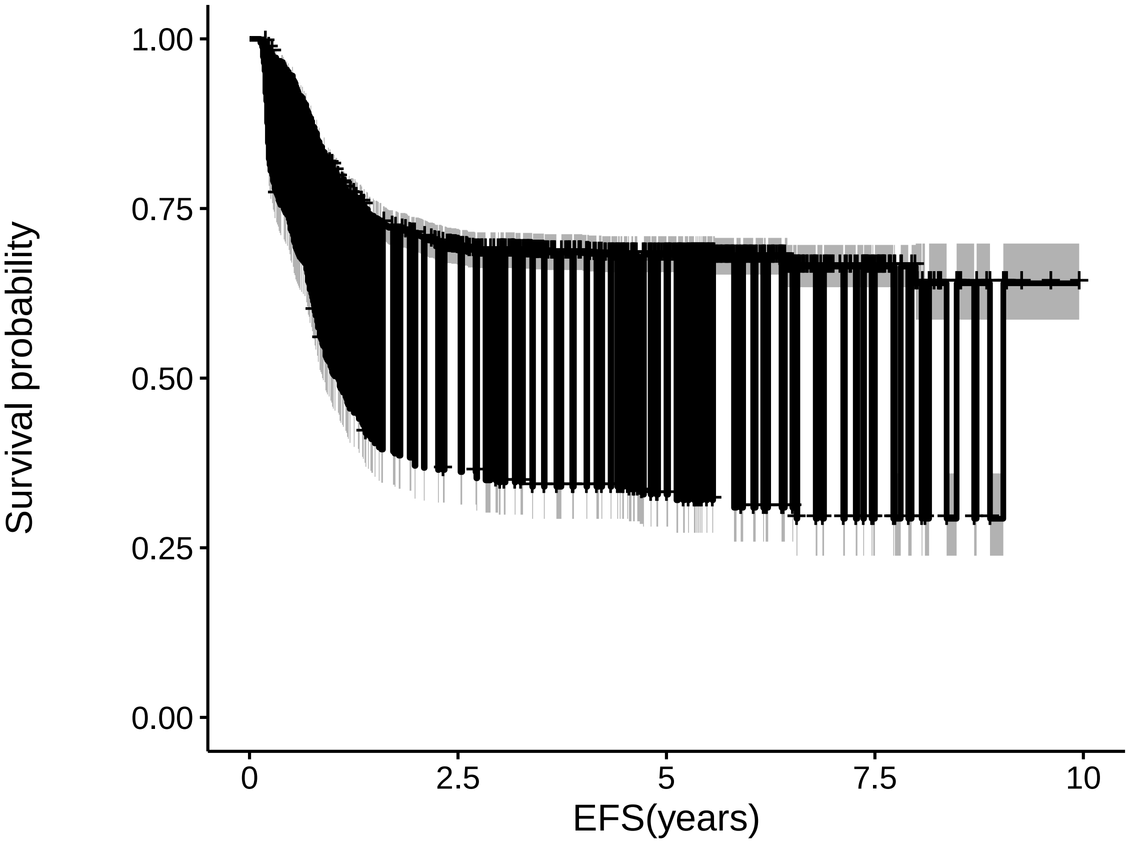


## Number at risk

| Strata | 0 | 2.5 | 5 | 7.5 | 10 |
|---|---|---|---|---|---|
| X23.CS=0 | 1203 | 793 | 595 | 56 | 0 |
| X23.CS=1 | 339 | 120 | 80 | 9 | 0 |

EFS(years)

## Number of censoring

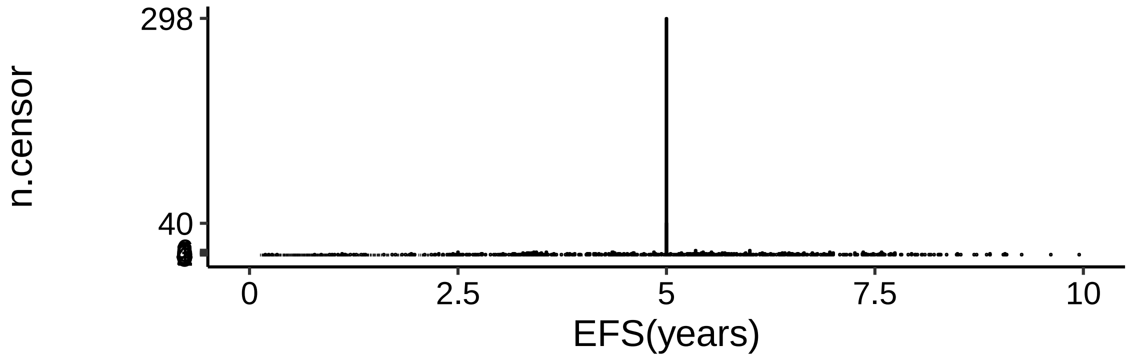

Supplement: S1 Code — (ZIP) [file pmed.1005088.s002.zip › S2 code/PROJ8_18_tbl2/PROJ8_18_tbl2_0.pdf]
